# The Amyloid-β-SDR5C1(ABAD) Interaction Does Not Mediate a Specific Inhibition of Mitochondrial RNase P

**DOI:** 10.1371/journal.pone.0065609

**Published:** 2013-06-05

**Authors:** Elisa Vilardo, Walter Rossmanith

**Affiliations:** Center for Anatomy and Cell Biology, Medical University of Vienna, Vienna, Austria; Oregon Health & Science University, United States of America

## Abstract

The amyloid-β peptide (Aβ) is suggested to cause mitochondrial dysfunction in Alzheimer’s disease. The mitochondrial dehydrogenase SDR5C1 (also known as ABAD) was shown to bind Aβ and was proposed to thereby mediate mitochondrial toxicity, but the molecular mechanism has not been clarified. We recently identified SDR5C1 as an essential component of human mitochondrial RNase P and its associated tRNA:m^1^R9 methyltransferase, the enzymes responsible for tRNA 5′-end processing and methylation of purines at tRNA position 9, respectively. With this work we investigated whether SDR5C1’s role as a subunit of these two tRNA-maturation activities represents the mechanistic link between Aβ and mitochondrial dysfunction. Using recombinant enzyme components, we tested RNase P and methyltransferase activity upon titration of Aβ. Micromolar concentrations of monomeric or oligomerized Aβ were required to inhibit tRNA 5′-end processing and position 9 methylation catalyzed by the SDR5C1-containing enzymes, yet similar concentrations of Aβ also inhibited related RNase P and methyltransferase activities, which do not contain an SDR5C1 homolog. In conclusion, the proposed deleterious effect of Aβ on mitochondrial function cannot be explained by a specific inhibition of mitochondrial RNase P or its tRNA:m^1^R9 methyltransferase subcomplex, and the molecular mechanism of SDR5C1-mediated Aβ toxicity remains unclear.

## Introduction

Alzheimer’s disease (AD) is the most common form of human dementia. Pathological hallmarks of the disease are amyloid plaques composed of the amyloid-β peptide (Aβ), neurofibrillary tangles of hyperphosphorylated tau protein and progressive neurodegeneration [1]. According to the “amyloid hypothesis”, the Aβ is considered the causative agent of the disease [2]; nevertheless, its role in the pathogenesis remains poorly understood. Aβs are natural products of cellular metabolism, ranging from 39 to 43 amino acids in length; Aβ_40_ is the most abundant form, whereas Aβ_42_ has a stronger propensity to aggregate. The overproduction of Aβ or the increased proportion of Aβ_42_ over Aβ_40_ appear sufficient to cause early onset AD [3]. An intracellular, mitochondrial pool of oligomeric Aβ was linked to impaired energy metabolism and increased production of reactive oxygen species, and Aβ-triggered mitochondrial dysfunction was suggested to be an early and possibly causative event in AD [4], [5].

Three mitochondrial proteins were reported to bind Aβ [6]–[8]. The best characterized is SDR5C1, a member of the short-chain dehydrogenase/reductase (SDR) family [9], [10], which was identified by yeast two-hybrid screening using Aβ as a bait. Due to an erroneous subcellular assignment, the protein was initially named endoplasmic reticulum-associated amyloid-β binding (ERAB) [6], but later renamed amyloid-β binding alcohol dehydrogenase (ABAD) [11]. SDR5C1 was reported to act on a wide range of substrates, such as fatty acids, hydroxysteroids, and alcohols, and it is essential for the degradation of isoleucine and short branched-chain fatty acids *in vivo* [12]. SDR5C1 binds Aβ with nanomolar affinity [6], [13], but a hundredfold higher concentration is required to inhibit its dehydrogenase activity [14], [15]. The Aβ-SDR5C1 interaction was also observed in coimmunoprecipitations from brain samples of deceased AD patients and a transgenic AD mouse model [16]. The inhibition of this interaction was reported to be protective against Aβ-mediated toxicity in cultured cells and mice [16], [17]. Nevertheless, how the impairment of SDR5C1’s dehydrogenase activity might link to mitochondrial dysfunction and Aβ-mediated toxicity remains unclear.

Recently we have shown that SDR5C1 is also an essential component of human mitochondrial RNase P (mtRNase P), a multifunctional enzyme complex responsible for 5′-end processing and methylation of mitochondrial tRNAs ((mt)tRNAs) [18], [19]. The mitochondrial genome is transcribed into long polycistronic precursor RNAs, comprised of 22 tRNAs interspersed among the rRNA and the protein-coding RNA sequences. MtRNase P is one of the enzymes responsible for the excision of tRNAs from these precursor transcripts [20]. It cleaves at the 5′ end of the tRNA structures and thus concomitantly releases the 3′ ends of rRNAs and mRNAs, all essential for mitochondrial translation and respiratory function. Moreover, a mtRNase P subcomplex containing SDR5C1 is responsible for the methylation of purines at position 9 of (mt)tRNAs [19], a modification essential for correct tRNA folding [21]. The knock down of SDR5C1 is sufficient to cause the accumulation of unprocessed tRNA precursors and to abolish methylation of (mt)tRNAs [18], [19].

Here we studied the effect of Aβ on the activity of human mtRNase P and its tRNA:m^1^R9 methyltransferase subcomplex, as a possible mechanistic link between the Aβ-SDR5C1 interaction and mitochondrial dysfunction.

## Results

### Soluble Aβ_42_ Oligomers Inhibit the Dehydrogenase Activity of SDR5C1

**Figure 1:**
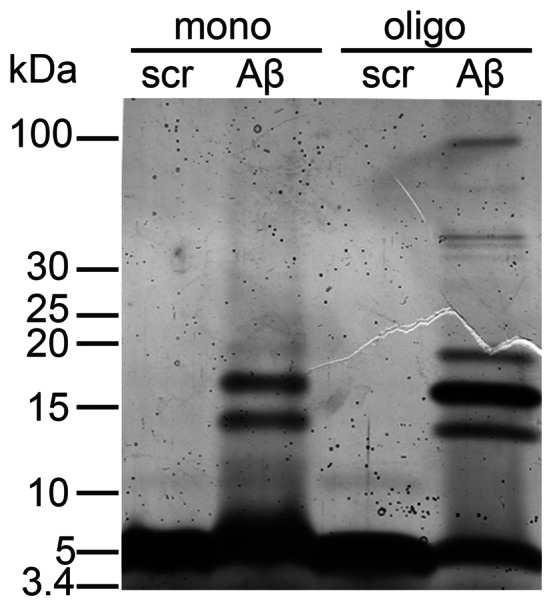
Characterization of Aβ_42_ preparations. Aβ_42_ and scrambled sequence peptide (scr) preparations were analyzed by 10–16% Tris·Tricine-SDS-PAGE and silver staining. 75 pmols each of freshly dissolved, mostly monomeric (mono) or “*in vitro* aged”, oligomeric (oligo) peptide were loaded. The molecular weight of a reference protein ladder is shown on the left.

To test the effect of Aβ on the various functions of SDR5C1, we used synthetic Aβ_42_ and a peptide of identical amino acid composition but scrambled sequence as a control. The attempt to isolate oligomeric forms from brain of AD individuals or mouse models, and to produce them *in vitro*, has led to a wealth of literature and confusing terminology referring to species ranging from dimers to more than 20-mers [22]. We employed a previously published protocol for “*in vitro* ageing” to obtain peptide preparations enriched in oligomers [23], and characterized them by polyacrylamide gel electrophoresis (PAGE). As shown in Figure 1, the control, scrambled sequence peptide migrated as a single band, compatible with the molecular weight of a monomeric peptide molecule (4.5 kDa), whereas Aβ_42_ showed two additional bands corresponding to trimeric and tetrameric forms, accounting for less than 10% of the total. The “*in vitro* aged” Aβ_42_ showed an enrichment of oligomers to more than 50%. The oligomeric forms ranged from trimers to ∼20-mers, with tetramers being the most conspicuous species (Fig. 1). The scrambled sequence peptide did not oligomerize.

**Figure 2:**
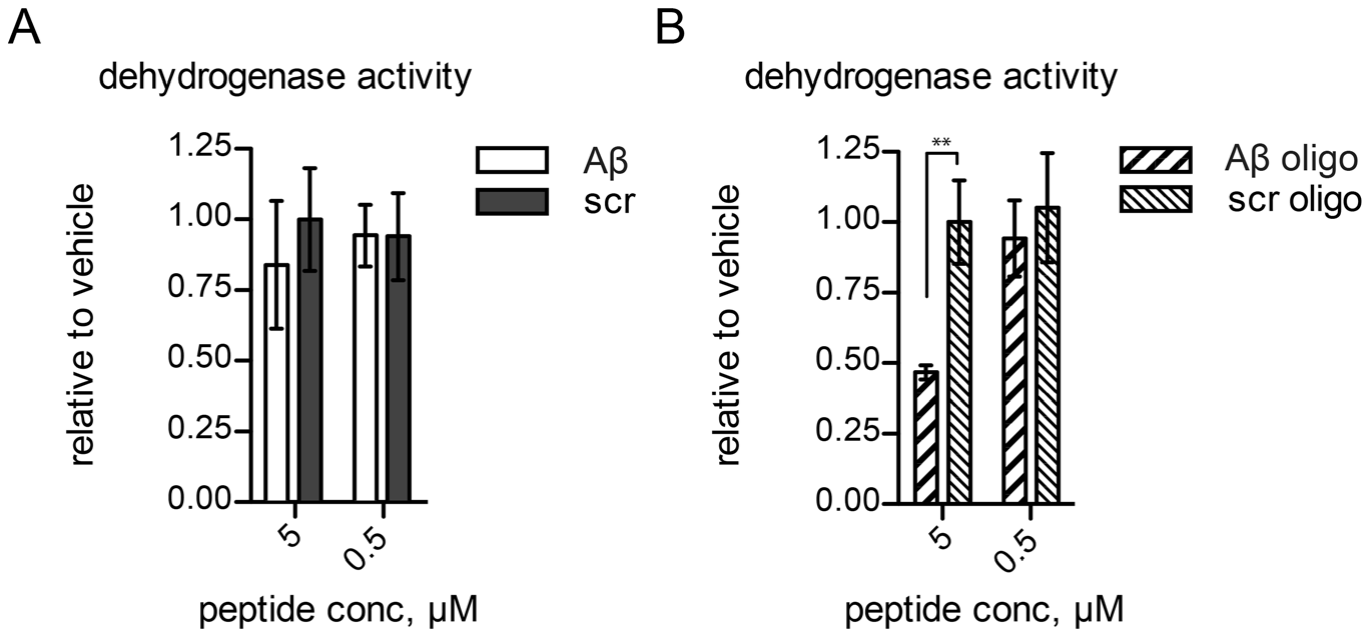
Effect of Aβ on the dehydrogenase activity of SDR5C1. L-3-hydroxyacyl-CoA dehydrogenase activity of SDR5C1 was measured with acetoacetyl-CoA as substrate and in presence of either (**A**) freshly dissolved or (**B**) “*in vitro* aged”, oligomeric (oligo) preparations of Aβ_42_ or scrambled sequence peptide (scr). Data are expressed relative to the activity of control reactions, to which only the solvent had been added. Mean and SD of two (A) and three (B) independent experiments are shown.

We assayed the dehydrogenase activity of purified SDR5C1 in presence of Aβ. The fresh, mostly monomeric Aβ_42_ had a negligible effect on the dehydrogenase activity of SDR5C1 (Fig. 2A), whereas the preincubation of the enzyme with 5 µM “*in vitro* aged”, oligomerized Aβ_42_ caused ∼50% inhibition (Fig. 2B). The scrambled sequence peptide had no effect on the dehydrogenase activity. These experiments are in agreement with the observation that only the oligomeric Aβ can bind SDR5C1 [13] and demonstrate the comparability of our peptide preparations to the ones previously employed to characterize the Aβ-SDR5C1(ABAD) interaction.

### Micromolar Concentrations of Aβ_42_ Inhibit RNase P Activity Independently of SDR5C1(ABAD)

**Figure 3:**
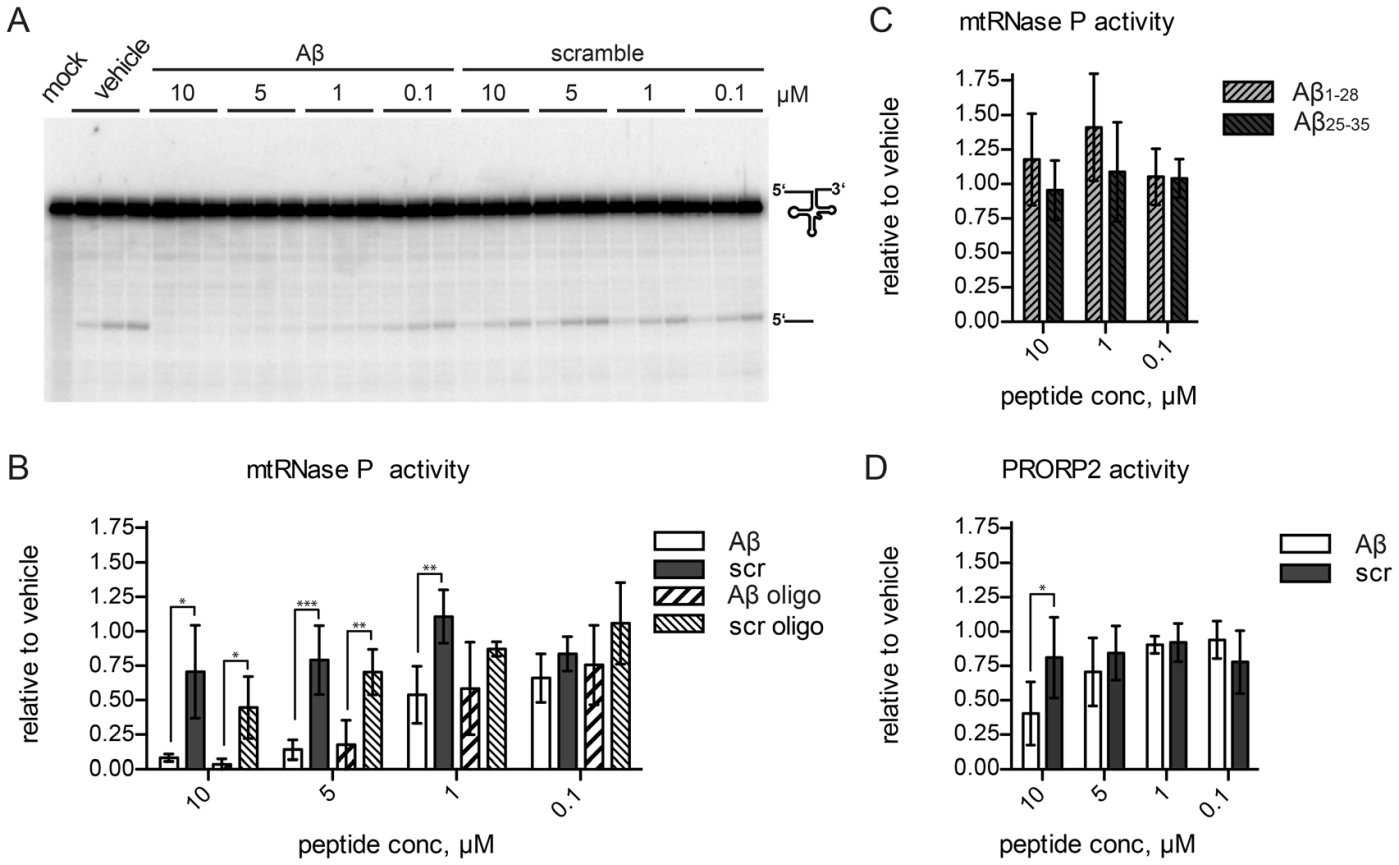
Effect of Aβ on RNase P activity. (**A**) A 5′-labeled (mt)tRNA^His^ precursor was cleaved with mtRNase P reconstituted from its recombinant components (TRMT10C-SDR5C1 and PRORP) in the presence of the indicated concentrations of freshly dissolved Aβ or scrambled sequence peptide. Reaction aliquots were withdrawn after 10, 30 and 60 minutes, stopped, and analyzed by denaturing PAGE and phosphor imaging. No enzyme was added to the mock reaction incubated for 60 minutes. The full length (mt)tRNA^His^ precursor and the released 5′ leader fragment are indicated on the right. (**B**) In experiments like that shown in (A), but with both, fresh and “*in vitro* aged”, oligomeric (oligo) peptide preparations, product formation was quantitatively analyzed and results plotted. Data are expressed relative to control reactions, to which only the solvent had been added. Mean and SD of three to five independent experiments are shown. (**C**) mtRNase P activity was assayed in the presence of freshly dissolved Aβ_1–28_ and Aβ_25–35_ fragments, and the results of three independent experiments were analyzed and plotted like in (B). (**D**) The RNase P activity of *A. thaliana* PRORP2 was assayed in the presence of different concentrations of fresh Aβ or scrambled sequence peptide and the results of four or five independent experiments were analyzed and plotted like in (B).

SDR5C1 together with TRMT10C and PRORP constitutes human mtRNase P [18]. We assayed the activity of the mtRNase P complex on a human (mt)tRNA^His^ precursor substrate in the presence of different concentrations of Aβ or control peptides. The titration of fresh or oligomerized Aβ_42_ inhibited the tRNA precursor cleavage with an IC_50_ of ∼2 µM, whereas the control peptide had only a negligible inhibitory effect (Fig. 3A and B). Similar results were obtained using (mt)tRNA^Ile^, (mt)tRNA^Lys^ and (mt)tRNA^Tyr^ (data not shown). It was previously reported that the part of Aβ corresponding to amino acids 1–20 mediates the binding to SDR5C1, while an Aβ fragment corresponding to amino acids 25–35 has no binding capacity [16]. We used the peptide fragments Aβ_1–28_ and Aβ_25–35_, but none of the two affected tRNA 5′-end processing by mtRNase P, even at the concentration of 10 µM (Fig. 3C).

In plants and some protists, nuclear and organellar RNases P consist of a single protein, homologous to human PRORP; unlike the human mitochondrial enzyme, they do not require any accessory protein [24]–[26]. When we tested the effect of Aβ_42_ on the tRNA 5′-end processing activity of *Arabidopsis thaliana* PRORP2, we observed inhibition at concentrations comparable to the one required to inhibit the human SDR5C1-containing mtRNase P enzyme (Fig. 3D and B). These results suggest that the inhibitory effect of Aβ on human mtRNase P activity is not mediated by SDR5C1, but caused by some unspecific effect of the peptide, as also indicated by the rather high concentrations of peptide required.

### Micromolar Concentrations of Aβ_42_ Inhibit tRNA Methyltransferase Activity Independently of SDR5C1(ABAD)

**Figure 4:**
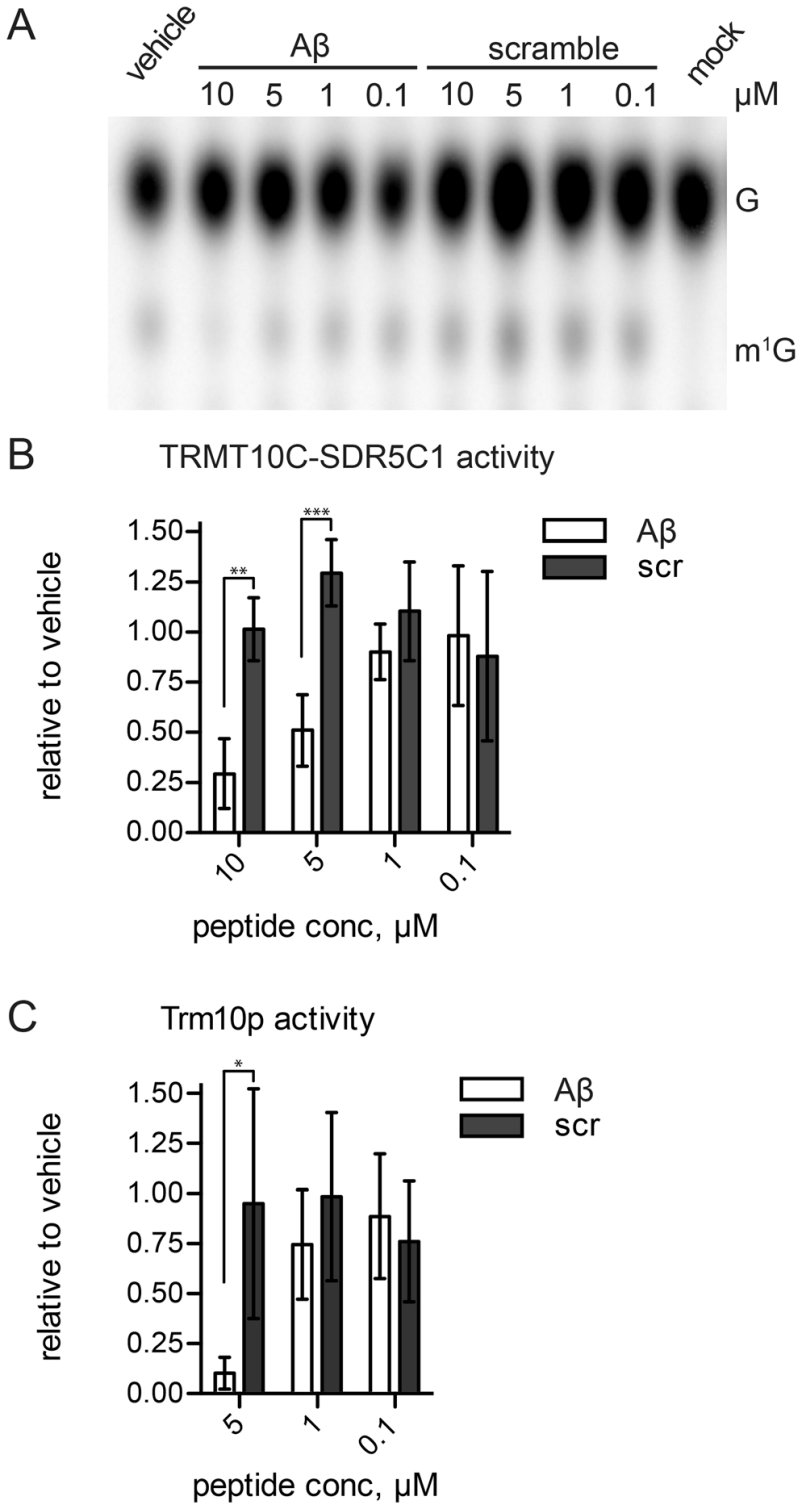
Effect of Aβ on tRNA:m^1^R9 methyltransferase activity. (**A**) (mt)tRNA^Ile^ specifically labeled at position 9 was incubated with the TRMT10C-SDR5C1 complex in the presence of a methyl group donor and the indicated concentrations fresh Aβ_42_ or scrambled sequence peptide. No enzyme was added to the mock reaction. The tRNA hydrolysate was resolved by TLC and visualized by phosphor imaging. The 30-minute time-point of the reactions is shown. (**B**) Product formation in experiments like that shown in (A) was quantitatively analyzed and results plotted. Data are expressed relative to control reactions, to which only the solvent had been added. Mean and SD of three or four independent experiments are shown. (**C**) The methyltransferase activity of yeast Trm10p was assayed in the presence of different concentrations of fresh Aβ or scrambled sequence peptide and the results of four independent experiments were analyzed and plotted like in (B).

The TRMT10C-SDR5C1 subcomplex of mtRNase P is a tRNA:m^1^R9 methyltransferase [19]. We tested whether Aβ affects the tRNA methylation mediated by the TRMT10C-SDR5C1 complex. Comparable to the effect on RNase P activity (Fig. 3A and B), micromolar concentrations of Aβ_42_ inhibited the methylation of the G at position 9 of (mt)tRNA^Ile^ (Fig. 4A and B). Similar results were obtained using (mt)tRNA^His^, a substrate that carries an A at position 9 (data not shown).

Trm10p, the TRMT10C homolog of *Saccharomyces cerevisiae*, catalyzes the methylation of G at position 9 of yeast tRNAs [27] and we recently showed that it is also active on human (mt)tRNAs *in vitro* [19]. In contrast to TRMT10C, the activity of yeast Trm10p does not depend on any accessory protein. We observed that the methyltransferase activity of Trm10p too was impaired by Aβ_42_ (Fig. 4C), further pointing to a generic rather than a specific mechanism of enzyme inhibition by Aβ_42_, unrelated to SDR5C1 or any specific Aβ-binding motif.

## Discussion

SDR5C1(ABAD) is proposed to be a crucial player in Aβ-induced mitochondrial dysfunction and, as a result, in AD [28]. However, the biological significance of the Aβ-SDR5C1 interaction and how it (mechanistically) links to mitochondrial dysfunction is largely unclear. SDR5C1 was reported to bind Aβ with a dissociation constant (K_d_) of ∼60 nM [6], [14], [16], but hundredfold higher concentrations of Aβ are required to inhibit its dehydrogenase activity ([14], [15] and this paper). Moreover, while SDR5C1 appears to be vital for mitochondrial function, this does not appear to be due to its dehydrogenase function; mitochondrial abnormalities associated with mutations in *HSD17B10* (the gene encoding human SDR5C1) do not seem to correlate with the residual dehydrogenase activity, suggesting that another function of SDR5C1 could actually be compromised and responsible for the mutation-associated neurodegenerative disease [29]. The discovery of SDR5C1’s essential role in tRNA maturation [18], [19] suggested a possible dehydrogenase-independent pathway leading from the interaction of Aβ with SDR5C1 to mitochondrial dysfunction. Specifically, we hypothesized that the binding of Aβ could impair the SDR5C1-dependent tRNA:m^1^R9 methyltransferase or mtRNase P activity.

Here we have shown that Aβ_42_, in its mainly monomeric as well as its oligomers-enriched form, inhibits the SDR5C1-associated tRNA:m^1^R9 methyltransferase and mtRNase P activity *in vitro*, but also that this inhibition of enzyme activity is an unspecific effect and is not mediated by the interaction of Aβ with SDR5C1. (i) Concentrations in the range of the reported dissociation constant did not have any effect on methylation or cleavage. Instead, micromolar amounts of Aβ were required to inhibit the tRNA:m^1^R9 methyltransferase and mtRNase P activity. Although Aβ was reported to accumulate in brain mitochondria of transgenic AD mice and AD patients [30], [31], it is unclear whether micromolar concentrations, able to inhibit the mitochondrial methyltransferase and RNase P, can be reached. (ii) Oligomers-enriched Aβ_42_ was not more potent in inhibiting the tRNA modification and processing activities than the freshly dissolved, mostly monomeric form, indicating that prior Aβ oligomerization is not required for inhibition. This contrasts with the observation that only oligomeric Aβ can bind SDR5C1 [13] and points to an SDR5C1 independent mechanism of inhibition. (iii) Neither Aβ fragment Aβ_1–28_, previously shown to be sufficient for SDR5C1-binding [16], nor Aβ_25–35_, inhibited the tRNA:m^1^R9 methyltransferase and mtRNase P activity. This finding too is consistent with an inhibitory mechanism of Aβ_42_ that is not mediated by SDR5C1. (iv) Finally, two related enzymes (yeast Trm10p and *A. thaliana* PRORP2), homologous to the respective catalytic subunits (TRMT10C and PRORP) but not associated with an SDR5C1-related protein, were inhibited by similar concentrations of Aβ. Altogether, these results suggest that a generic, rather than a specific mechanism is underlying the inhibition of tRNA methylation and cleavage, and the high concentration of Aβ required suggests that it is likely not physiologically relevant.

In conclusion, the proposed deleterious effect of Aβ on mitochondrial function cannot be explained by an inhibition of human mtRNase P or its tRNA:m^1^R9 methyltransferase subcomplex and the molecular mechanism of SDR5C1-mediated Aβ toxicity remains unclear.

## Materials and Methods

### Expression and Purification of Recombinant Proteins

We used N-terminally His-tagged SDR5C1, native (untagged) TRMT10C, and C-terminally His-tagged human PRORP and *A. thaliana* PRORP2, all described previously [18], [19], [32]. The plasmid for the expression of yeast Trm10p was kindly provided by Jane Jackman [27]. Proteins were expressed in *E. coli* and purified as described previously [18], [19], [32]. Briefly, bacteria were broken by sonication and His-tagged proteins purified by affinity chromatography. Purified SDR5C1 was mixed with a crude bacterial lysate of native TRMT10C and the TRMT10C-SDR5C1 complex purified on a HisTrap HP column. Protein concentrations were calculated from the absorbance at 280 nm, molar extinction coefficient and molecular weight.

His-tagged and native SDR5C1 have nearly identical affinity for Aβ_42_ [6], [16], and neither dehydrogenase activity nor RNase P or methyltransferase activities are impaired by the N-terminal His-tag [15], [18], [19], [33].

### Peptide Preparation and Characterization

Aβ_42_, Aβ_1–28_, Aβ_25–35_ and the scrambled sequence peptide were of synthetic origin (rPeptide). Peptides were dissolved in 1,1,1,3,3,3-hexafluoro-2-propanol (HFIP) at 5 mg/ml by shaking at room temperature for 1 hour [34]. The peptide solutions were aliquoted and HFIP was removed by evaporation in the fume hood and then in a SpeedVac. Peptide aliquots were stored desiccated at −80°C.

Immediately before use, an aliquot of peptide was thawed, redissolved in 100% DMSO to 5 mM concentration, sonicated for 5 minutes and further diluted in assay buffer. Peptide quality was routinely assessed by dilution in 2× Tricine loading buffer (100 mM Tricine, 100 mM Tris·Cl pH 6.8, 24% Glycerol, 8% SDS, 0.02% Coomassie brilliant blue G-250) and separation by 10–16% Tris·Tricine-SDS-PAGE [35], followed by silver staining. The observed band pattern was identical for the three different peptide batches used in this study.

“*In vitro* aged” peptide was prepared by SDS-induced oligomerization [23]. Briefly, an aliquot of peptide was incubated at the concentration of 400 µM in 0.2% SDS in PBS, at 37°C for 6 hours, then diluted with 3 volumes of H_2_O and incubated for further 18 hours at 37°C. The peptide was subsequently precipitated with a ninefold excess (v/v) of ice-cold methanol/acetic acid and redissolved in 35 mM NaCl, 5 mM NaH_2_PO_4_ pH 7.4. The treatment led to the reproducible enrichment of soluble Aβ_42_ oligomers with minor peptide loss, as assessed by Tris·Tricine-SDS-PAGE. Concentrations of “*in vitro* aged” peptide were estimated by Tris·Tricine-SDS-PAGE and silver staining relative to fresh Aβ_42_, and expressed in terms of Aβ_42_ monomer equivalents. The scrambled sequence peptide was treated in the same way for use as a control in inhibition experiments, but did not show any oligomerization.

### Dehydrogenase Assay

l-3-hydroxyacyl-CoA dehydrogenase activity was measured as acetoacetyl-CoA dependent NADH dehydrogenation [36].

### RNase P Assay

*In vitro* transcription, ^32^P 5′ end labeling and purification of the (mt)tRNA^His^ precursor substrate, and RNase P activity assays were carried out and analyzed as described previously [18], [19] with the following changes. Trace amounts of radioactively labeled substrate with unlabeled substrate at a final concentration of 250 nM were cleaved with 50 nM reconstituted, recombinant mtRNase P or *A. thaliana* PRORP2 at 21°C in 50 mM Tris·Cl pH 8, 20 mM NaCl, 4.5 mM MgCl_2_, 1 mM DTT, 20 µg/ml BSA, 20 units/ml RNase inhibitor. Samples for analysis were withdrawn at defined intervals or at known linear-range time-points and phosphor imaging data quantitatively analyzed with ImageQuant TL 7 (GE Healthcare).

### Methyltransferase Assay

*In vitro* transcription, internal ^32^P labeling at position 9 and purification of the (mt)tRNA^Ile^ substrate, and methyltransferase assays were carried out as described previously [19], with the following changes. Trace amounts of radioactively labeled substrate with unlabeled substrate at a final concentration of 250 nM were incubated in the above-specified reaction buffer, with 25 µM *S*-adenosyl methionine and 50 nM TRMT10C-SDR5C1 complex or yeast Trm10p at 30°C. Samples for analysis were withdrawn at defined intervals or at known linear-range time-points and phosphor imaging data quantitatively analyzed with ImageQuant TL 7 (GE Healthcare).

### Statistical Analysis

Aβ-treated and scrambled sequence peptide-treated enzyme activities were compared by the unpaired, two-tailed t test (*, P<0.05; **, P<0.01; ***, P<0.001).
